# Supplementary figures and images for: SARS-CoV-2 variants of concern Alpha and Delta show increased viral load in saliva
Source: PLoS One. 2022 May 10;17(5):e0267750. doi: 10.1371/journal.pone.0267750 (PMC9089873; doi:10.1371/journal.pone.0267750)

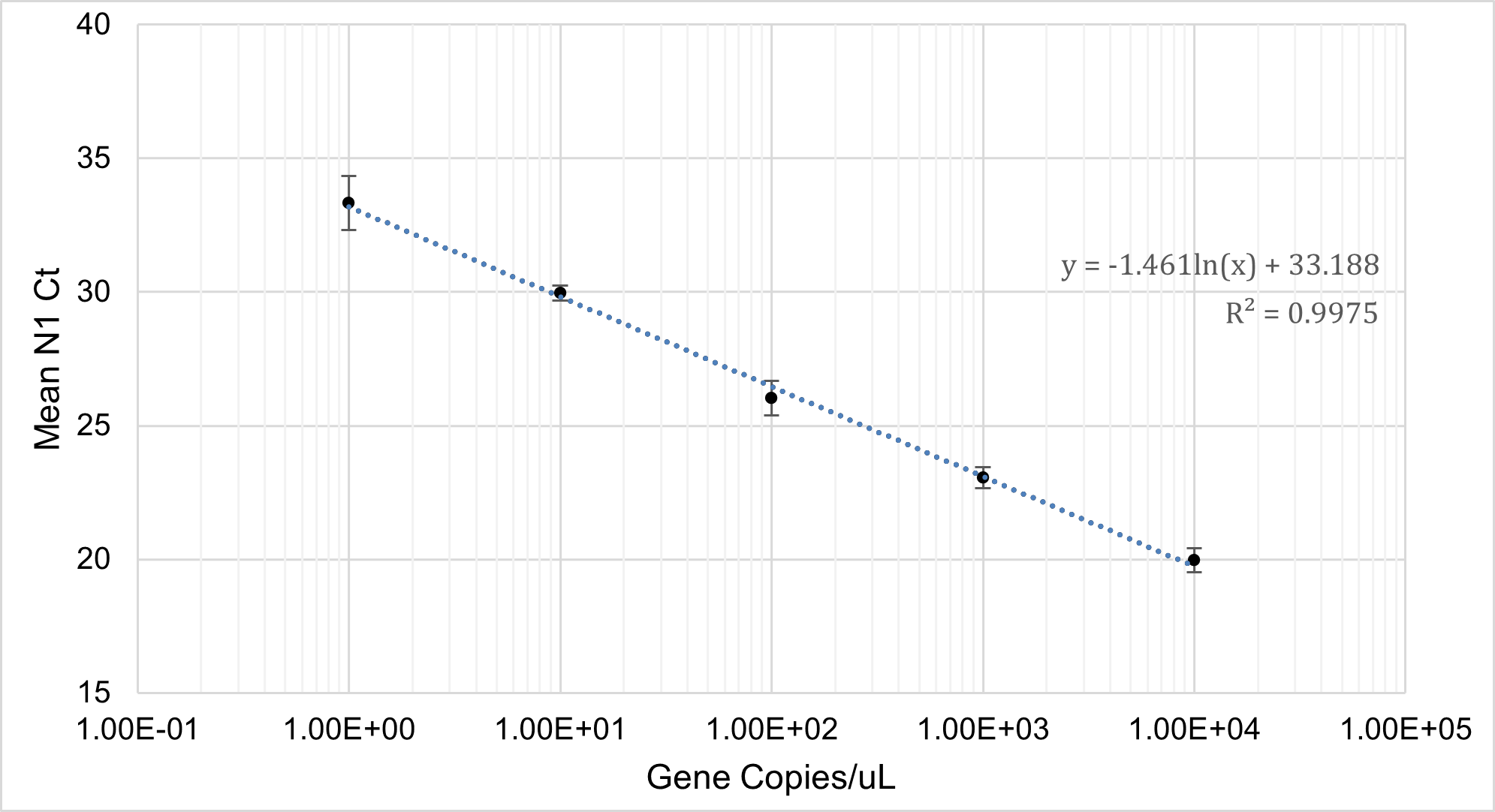

Supplement: S1 Fig — The standard curve was plotted with standard deviations to determine the range of accurate detection using this primer/probe combination. The mean Ct values (n = 4) obtained from serial dilutions were plotted against estimated quantify of synthetic RNA in 10μL of RT-qPCR reaction. (TIF) [file pone.0267750.s001.tif]

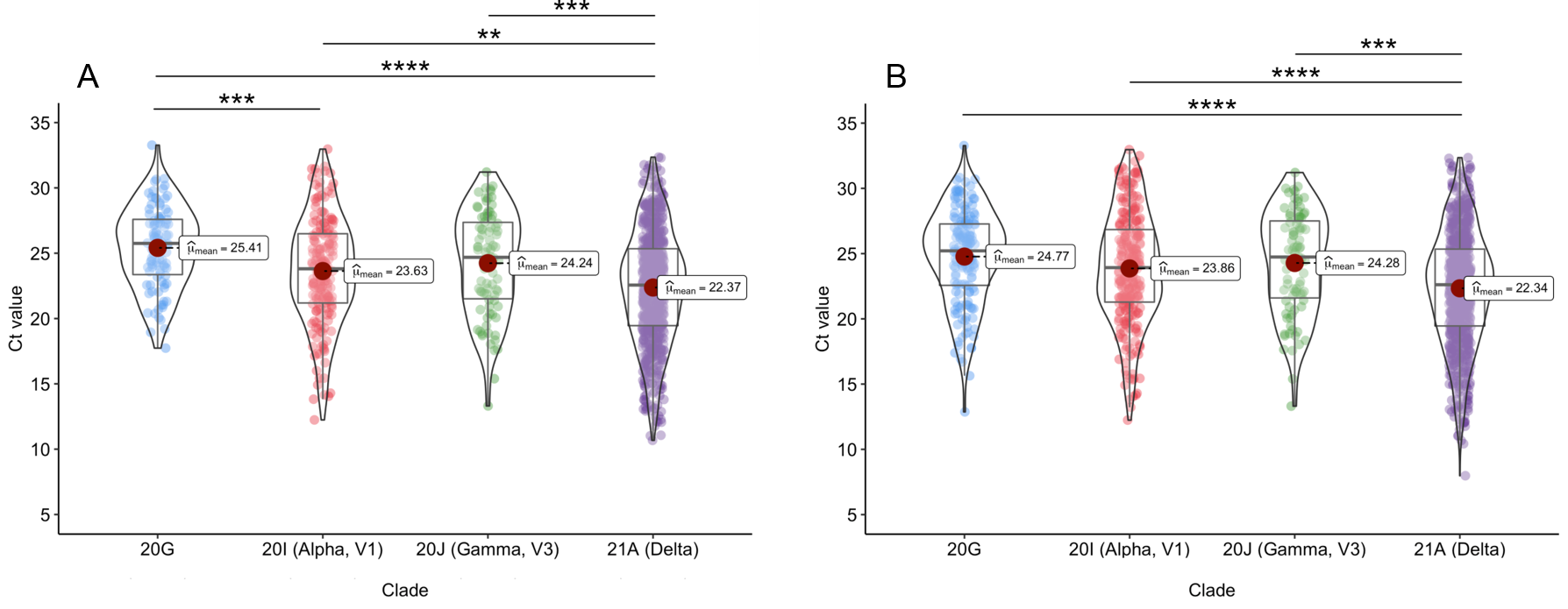

Supplement: S2 Fig — 2A: Comparison of all samples. We observed a statistically significant difference between Delta and all other clades, including an 8-fold difference in viral load when compared to 20G. 2B: Comparison of only surveillance samples. The same difference in median Ct was observed between Delta and all other clades. Additionally, surveillance samples showed a statistical difference between Alpha and 20G. *p.adj<0.05, **p.adj<0.01, ***p.adj<0.001, ****p.adj<0.0001. (TIF) [file pone.0267750.s002.tif]
