# Supplementary material for: SARS-CoV-2 variants of concern Alpha and Delta show increased viral load in saliva
Source: PLoS One. 2022 May 10;17(5):e0267750. doi: 10.1371/journal.pone.0267750 (PMC9089873; doi:10.1371/journal.pone.0267750)
Supplement: S1 File — (DOCX) [file pone.0267750.s007.docx]

**Supplemental Information:**

List of reagents for diagnostic assay:

| Reagent | Vendor | Part Number | Description |
| --- | --- | --- | --- |
| HS_RPP30 Synthetic DNA | Integrated DNA Technologies | 299788131 | P1 positive control |
| Luna Buffer Probe One-Step Reaction | New England Biolabs | M3006B |  |
| Luna WarmStart RT Enzyme Mix | New England Biolabs | M3002B |  |
| nCOV_N1 Probe Aliquot, 50 nmol | Integrated DNA Technologies | 10006832 | FAM-ACC CCG CAT /ZEN/ TAC GTTTGGTGGACC-3IABkFQ |
| nCOV_N1 Forward Primer, 100 nmol | Integrated DNA Technologies | 10006830 | GACCCCAAAATCAGCGAAAT |
| nCOV_N1 Reverse Primer, 100 nmol | Integrated DNA Technologies | 10006831 | TCTGGTTACTGCCAGTTGAATCTG |
| RNAse P (ATTO 647) Probe, 50 nmol | Integrated DNA Technologies | 10007062 | 5Cy5-TTC TGA CCT /ZEN/ GAA GGCTCTGCGCG-3IABkFQ |
| RNAse P Forward Primer, 100nmol | Integrated DNA Technologies | 10006836 | AGATTTGGACCTGCGAGCG |
| RNAse P Reverse Primer, 100 nmol | Integrated DNA Technologies | 10006837 | GAGCGGCTGTCTCCACAA GT |
| Sars-CoV-2 Synthetic RNA Control 2 | Twist Biosciences | 102024 / 103907 / 103909 | N1 Positive Control |


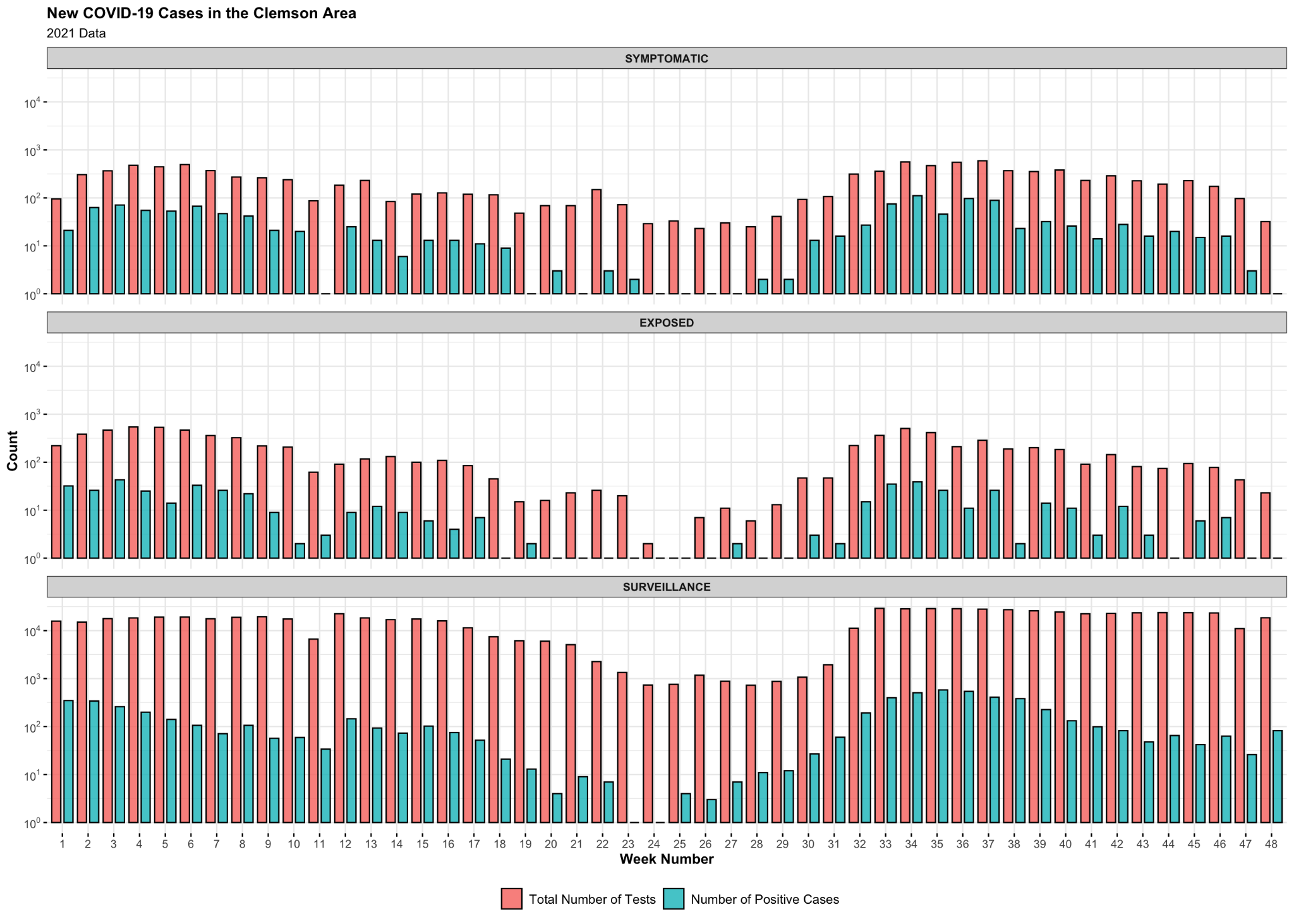


**Supplemental Figure 2:** Number of tests and positive tests per category, by week. Note that the y-axis is on a log10 scale. Samples are labeled “symptomatic” if the patient reports symptoms at the time of testing, or labeled “exposed” if they report exposure to a positive patient. Surveillance samples represent the rest of the samples collected. The lower case load during week 11 is due to the university’s spring break, and weeks 18-29 account for summer break.


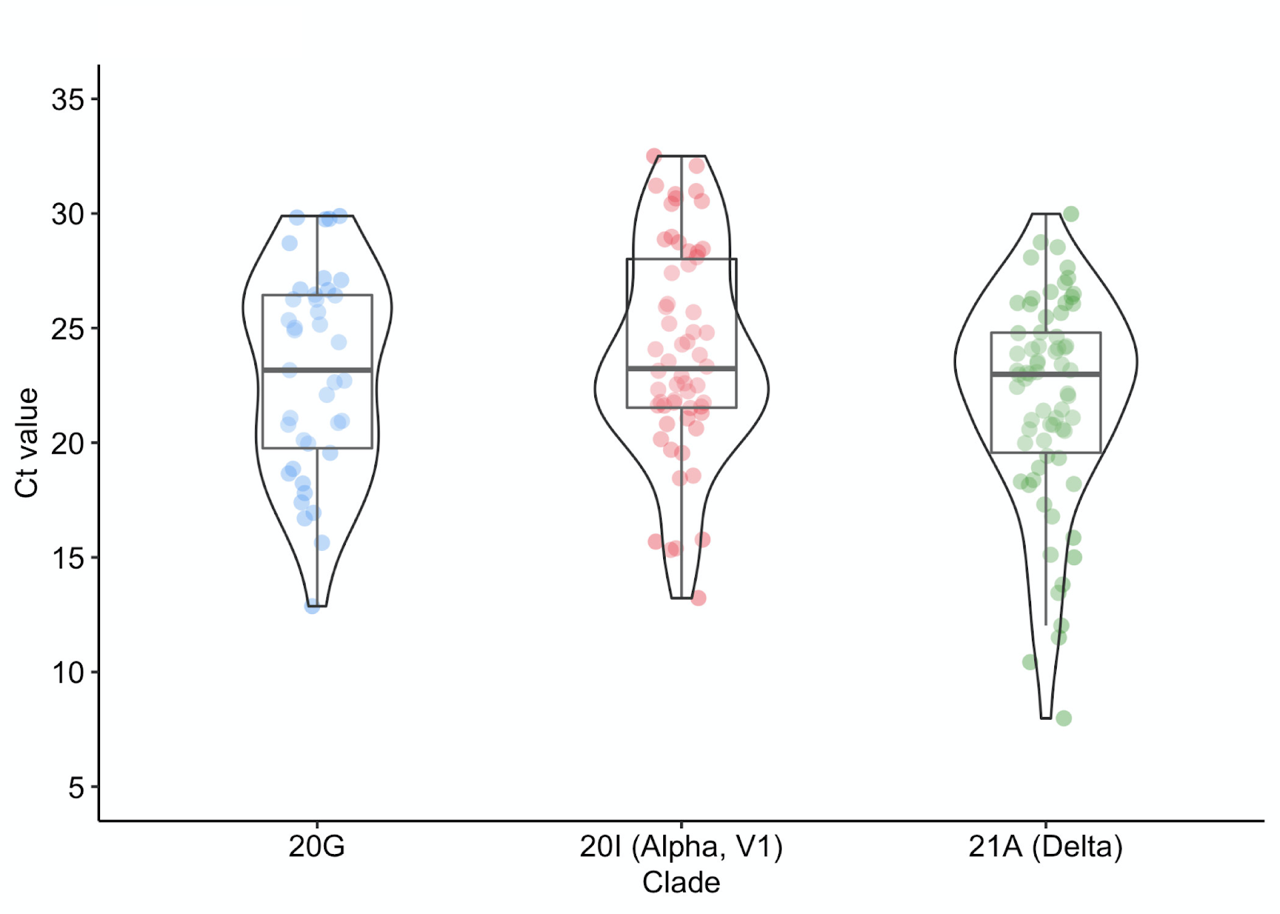


**Supplemental Figure 3**: Comparison of N gene Ct values among symptomatic samples for 20G (n=39), Alpha (n-58), and Delta (n=10). Using the Kruskal-Wallace test, no statistically significant difference was observed between Delta [22.985 (7.980-29.980)] and all other clades [20G: 23.165 (12.870-29.890; Alpha: 23.230 (13.225-32.505)].
